# Brain Activation Features in Response to the Expectation of Receiving Rewards Through Aggression

**DOI:** 10.3390/brainsci15121326

**Published:** 2025-12-12

**Authors:** Jia-Ming Wei, Xiaoyun Zhao, Ling-Xiang Xia

**Affiliations:** 1 College of Education, Huaibei Normal University, Huaibei 235000, China; weijm@chnu.edu.cn (J.-M.W.); xiaoyunzhao@chnu.edu.cn (X.Z.); 2 Research Center of Psychology and Social Development, Faculty of Psychology, Southwest University, Chongqing 400715, China; 3 Key Laboratory of Cognition and Personality, Ministry of Education, Southwest University, Chongqing 400715, China

**Keywords:** reward expectation, aggression, moral, brain correlates, Harm–Gain Task, fMRI

## Abstract

**Background**: Reward expectation is an important motivation for aggression. However, despite substantial progress in behavioral studies related to reward expectation in aggression, the neural basis underlying this process remains unclear. **Methods**: To investigate the brain correlates of aggressive reward expectation, we developed the Harm–Gain Task (HGT). In this task, participants were informed that they could gain money by causing harm to another person and were instructed to evaluate their satisfaction with the anticipated monetary reward. Additionally, we designed a questionnaire to measure participants’ moral disengagement concerning aggressive decision-making in the HGT. Thirty-four healthy Chinese university students completed the HGT while in the scanner, and their functional images were acquired using a 3.0-T Siemens Tim Trio scanner. Data from two participants were excluded from the analysis due to excessive head motion. Finally, data from 32 participants (15 males, *M*_age_ = 19.97 years, *SD*_age_ = 2.07 years) were included in the analyses. **Results**: Findings show that during the reward expectation phase of the HGT, (1) relative to the baseline condition, the orbitofrontal cortex (OFC), anterior cingulate cortex (ACC), and middle cingulate cortex (MCC) were significantly activated. Conversely, activation in the bilateral dorsolateral prefrontal cortex (DLPFC), bilateral inferior parietal lobule (IPL), and bilateral lateral temporal cortex (LTC) was attenuated. (2) As the monetary amount raised, activation in the OFC and ACC significantly increased, while activation in the DLPFC, IPL, and LTC significantly decreased. (3) As the monetary amount raised, the heightened activation in the OFC and ACC was significantly correlated with participants’ aggressive behavior and moral disengagement scores. **Conclusions**: The results provide preliminary evidence regarding neural correlates in aggressive reward expectation, promoting further exploration of the cognitive neural mechanisms underlying aggression.

## 1. Introduction

Expecting potential rewards or gains is a crucial influencing factor on human behavior [1,2]. In aggression research, reward expectation is a critical cognitive factor promoting aggressive behavior [3,4,5] and is considered reinforcement-based motivation for aggression [6]. Previous studies have indicated that reward expectation can predict various forms of aggressive behavior, including bullying [7], intimate partner violence [8], and workplace aggression [9]. Moreover, it is also related to psychological health problems such as depression, trauma, and emotional disorders [8]. Therefore, research on reward expectation in aggressive behavior is both necessary and valuable. Referring to previous studies [5,10,11], in the present study, we define aggressive reward expectation as the cognitive process of anticipating desired outcomes gained by harming others.

However, although significant progress has been made in the behavioral study of aggressive reward expectation, research on its neural mechanisms remains inadequate. The brain serves as a crucial neural basis for aggression, and task-based functional Magnetic Resonance Imaging (fMRI) provides an effective means to investigate the brain correlates of aggressive cognitive mechanisms [12,13]. Therefore, to uncover the principles and neural mechanisms of aggressive reward expectation, this study employed fMRI to investigate its brain correlates.

Although the evidence regarding the neural mechanisms of aggressive reward expectation is insufficient, it is possible to infer its brain correlates based on the associated mental components. Based on previous theories and research findings [5,13,14,15,16], we propose that the process of reward expectation in aggressive behavior is closely linked to the following mental components: episodic future thinking, reward processing, moral inhibition, and moral conflict.

First, aggressive reward expectation involves envisioning and speculating about the potential benefits that aggression might yield. Episodic future thinking, a form of prospection [16], represents imaging or simulating experiences that might occur in one’s personal future [17]. In other words, episodic future thinking likely constitutes a fundamental mental component of aggressive reward expectation. Previous studies indicate that the default network serves as an important brain substrate in representing imagined future events and behavioral outcomes [17,18,19]. Specifically, the medial temporal lobe system within the default network, comprising the hippocampus, retrosplenial cortex, ventral medial prefrontal cortex, and posterior inferior parietal cortex, is primarily involved in simulating future episodic events. The dorsal medial prefrontal cortex (dMPFC) subsystem, which includes the dMPFC, temporoparietal junction, and lateral temporal cortex, mainly relates to self-concerned cognition, such as inferring one’s mental states in future episodic events [18,19,20,21]. In summary, the default network is likely linked to aggressive reward expectation.

Secondly, reward processing is the core mental component in aggressive reward expectation. Many research findings indicated that the striatum (including the putamen, caudate, and nucleus accumbens) [22,23] and orbitofrontal cortex (OFC) [24,25,26] are closely related to the cognitive representation of expected material or social rewards. The striatum is associated with the “action–outcome” cognitive association [27]. When cues predicting reward are presented, or individuals imagine positive behavioral outcome, the striatum shows significant activation [22,23,27]. Additionally, the OFC is critical for the subjective value representation of expected reward [26,28,29,30]. When individuals assign a higher subjective value to expected reward [24,31,32] or anticipate receiving desired reward [33,34], the activation level of the OFC significantly increases. Moreover, the striatum is linked to the motivational salience [23] and the OFC encodes the motivational value of anticipated reward [26,35]. In other words, the striatum and OFC may be associated with the incentive effect of reward expectation on aggressive behavior [13]. To sum up, the striatum and OFC may be play critical roles as brain correlates of aggressive reward expectation.

Thirdly, the process of aggressive reward expectation may involve moral inhibition and moral conflict. According to Cognitive Neoassociation Theory [36,37], the activation of aggression-related mental components is diffuse and interconnected. Under the influence of expected reward, individuals may have aggressive intentions or inclinations [38,39] that contradict moral standard and social norm [40,41]. In this case, morally relevant schemas, such as moral inhibition, may also be activated [13,39]. In other words, during aggressive reward expectation, a moral conflict may emerge between aggressive inclination and the moral inhibition. Therefore, brain regions associated with moral inhibition and moral conflict are likely to be involved in the neural correlates of aggressive reward expectation. Research in neuroethics demonstrated the involvement of the dorsolateral prefrontal cortex (DLPFC) in moral judgment, while the inferior parietal lobule (IPL) and insula are associated with moral emotions (e.g., empathy, guilt) [42,43]. Additionally, the anterior cingulate cortex (ACC) is a critical brain region for conflict processing, including conflict monitoring, resolution, and regulation, and plays a role in the trade-off between behavioral costs and benefits [13,44,45,46,47]. A recent fMRI study observed significant activation in the ACC during aggressive decision-making which involved monetary incentives and moral inhibition [13]. In summary, the DLPFC, IPL, insula, and ACC may be closely associated with aggressive reward expectation.

The principal aim of our study is to explore the brain correlates of aggressive reward expectation. For this purpose, we developed the Harm–Gain Task (HGT) with reference to previous research [13,39]. In the HGT, participants can gain money by administering uncomfortable electrical stimulation to another individual. When informed about the amount of money that can be gained through causing harm, participants are required to assess to what extent the reward satisfies them. Following the assessment, participants decide whether or not to harm another person in order to receive money. The HGT comprises two experimental conditions: high monetary and low monetary.

In summary, we propose that episodic future thinking, reward processing, moral inhibition, and moral conflict are fundamental mental components of aggressive reward expectation. The brain regions associated with these components may constitute the brain correlates of aggressive reward expectation. Therefore, we propose the following hypotheses:

The first hypothesis is that, in the HGT, significant activation differences between the aggressive reward expectation and the baseline condition can be observed in the OFC, striatum, ACC, DLPFC, IPL, insula, and regions within the default network.

The second hypothesis is that, in the HGT, during the reward expectation phase, relative to the low monetary condition, the activation in brain regions related to reward processing (e.g., OFC, striatum) and moral conflict (e.g., ACC) significantly increases under the high monetary condition. Conversely, activation in brain regions associated with moral inhibition, such as the DLPFC and IPL, decreases.

Moreover, according to the aggression motivation theory, expected reward, serving as a driving force, could induce individuals to generate aggressive intentions that conflict with moral standard [5,13]. To avoid moral conflict and facilitate aggressive behavior, individuals may employ moral disengagement [5,48], a cognitive strategy that reconstructs immoral behavior as morally acceptable [40,49]. In other words, moral disengagement may reflect a cognitive manifestation of moral conflict that individuals undergo, and reward expectation for aggression is a prerequisite for employing moral disengagement [5]. Therefore, the OFC, which is involved in the motivational value of expected reward [26,35], may be related to participants’ aggressive responses and moral disengagement thoughts in the HGT. On the other hand, the ACC is critical in processing cognitive conflicts [13,46]. In the HGT, activation in the ACC may indicate the conflict between aggressive inclinations and moral inhibition during the aggressive reward expectation phase. To alleviate the negative moral conflict state, participants may engage in moral disengagement. Thus, in the HGT, ACC activation during aggressive reward expectation is likely related to moral disengagement thoughts regarding harming others. In conclusion, we have presented two further hypotheses:

The third hypothesis is that, in the HGT, during the reward expectation phase, relative to the low monetary condition, activation in the OFC under the high monetary condition significantly correlates with participants’ aggressive behavior and moral disengagement scores.

The fourth hypothesis is that, in the HGT, during the reward expectation phase, relative to the low monetary condition, activation in the ACC under the high monetary condition significantly correlates with participants’ moral disengagement scores.

## 2. Materials and Methods

**Figure 1:**
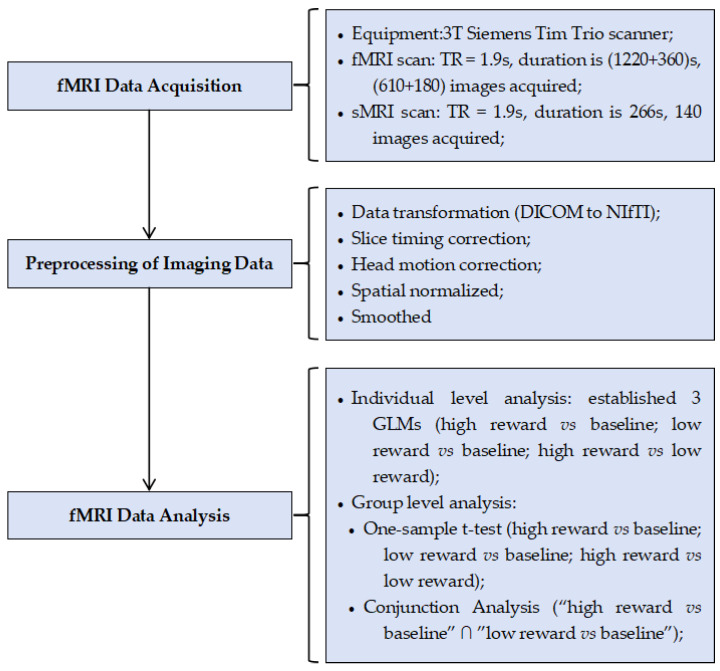
Structural diagram of the fMRI approach employed in the present study.

The study used a within-subject, repeated-measures design. Specifically, participants performed the HGT inside an MRI scanner to examine brain activation patterns during aggressive reward expectation and assess the differences in brain activation across various reward expectation conditions. The primary purpose of this study was to use fMRI to explore the neural correlates of aggressive reward expectation and its predictive effects on aggression and state moral disengagement in the HGT. The fMRI data acquisition and analysis pipeline is depicted in Figure 1.

### 2.1. Participants

Healthy participants who met the following criteria were included in this study: right-handed, normal vision (or corrected to normal), no history of neurological or psychiatric disorders, no metal implants (e.g., medical devices) in the body, and no claustrophobia. Participants whose head motion during scanning was excessive (i.e., translational parameters > 3 mm or rotational parameters > 3 rad) were excluded from the subsequent analysis.

Participants were recruited through a convenience sampling method. Specifically, advertisements were posted both online (e.g., on social media platforms) and offline (e.g., on paper flyers), which included information about the study and the compensation participants would receive. University students who showed interest in the study were allowed to apply for the experiment. After a review process (e.g., screening for metal implants), the researchers determined whether the applicants were eligible to participate. Thirty-four healthy Chinese university students were recruited. All participants gave informed consent, and two participants were excluded because of excessive head motion. Consequently, 32 participants (15 males, 19–29 years old, *M*_age_ = 19.97 years, *SD*_age_ = 2.07 years) remained.

The study was approved by the Research Project Ethical Review Committee of the Faculty of Psychology at Southwest University (approval number: H22043). After obtaining written consent from participants, all were compensated with money for participation.

### 2.2. The Harm–Gain Task (HGT)

The HGT consisted of Role A and Role B, who were of the same sex. Role A had the option to receive money by administering electric stimulation to Role B’s left wrist. If Role A refused to administer electric stimulation to Role B, they would not receive any money. Role B could only passively accept Role A’s decision and could not retaliate against Role A. Before initiating the task, all participants had to draw their roles. Participants were informed that the experimenter randomly assigned their roles. In fact, all participants were assigned the identity of Role A. Role B was enacted by the experimenter’s assistant and was exempt from receiving electric stimulation.

After the participants drew their identities, the experimenter administered electric stimulation to their left wrist for 0.5 s per stimulation. The electric stimulation started with a low intensity and was gradually increased. Participants were required to verbally report their discomfort levels when receiving electrical shocks. The experimenter provided the participants with a 9-point scale, where 1 indicated “no discomfort,” and 9 indicated “marked discomfort,” with higher numbers indicating greater discomfort. When participants reported a rating of “9,” the experimenter terminated the electric stimulation. Then, participants were informed that the subjective level of discomfort experienced by Role B during the electric stimulation was also rated as “9.” This procedure’s purpose is to allow participants to experience the intensity of harm inflicted on Role B. The electrical stimulation intensity used in HGT was strictly controlled within a safe range (maximum ≤ 10 mA), posing no risk of physical injury.

**Figure 2:**
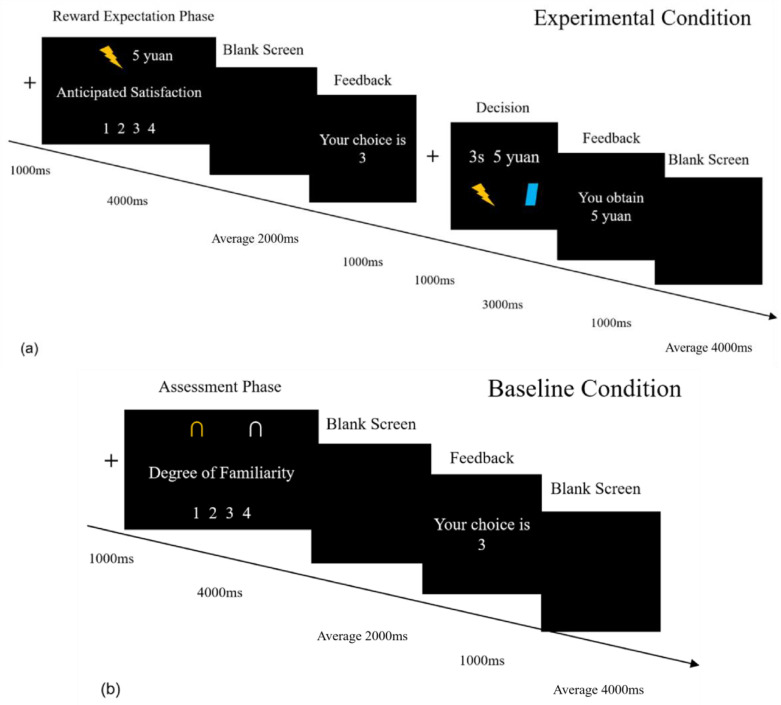
(**a**) An example of trial time course in the HGT. (**b**) An example of trial time course in the symbol assessment task. The word “yuan” refers to the Chinese yuan (CNY).

The HGT consisted of two experimental conditions, each comprising 30 trials (see Figure 2 for an example). In the high monetary condition, participants could receive rewards ranging from CNY 13 to 20 by administering electric stimulation to Role B. In the low monetary condition, participants could receive monetary rewards ranging from CNY 1 to 8 by administering electric stimulation to Role B. Each trial began with a fixation presented for 1 s, followed by the reward expectation phase. During the reward expectation phase, participants were informed of the amount of money they could gain by administering electric stimulation (represented by a lightning symbol) to Role B. They were also required to assess their anticipated satisfaction for gaining money by harming Role B. They could use keys 1, 2, 3, and 4 to make their assessment, with 1 indicating “not satisfied” and 4 indicating “very satisfied,” where higher numbers corresponded to greater satisfaction. Participants had up to 4 s to perform the assessment. Subsequently, a blank screen (with an average duration of 2 s) was presented, followed by feedback on the participant’s assessment. In the following phase, participants were informed about the duration of electric stimulation they were allowed to apply to Role B. They were required to determine whether to harm Role B for money. Participants were given a maximum of 3 s to make their decision. After the participants’ decision-making, feedback was provided. Each trial ended with a blank screen (with an average duration of 4 s), indicating the start of the next trial. Additionally, 10 extra experimental trials were added as fillers and were not included in the data analysis. The presentation of all trials was randomized.

Lastly, participants were required to complete a symbol assessment task consisting of 30 trials. They were instructed to assess their familiarity with certain neutral symbols (e.g., ∩, a mathematical symbol) presented in this task. The fMRI data from the assessment phase serve as a baseline for data analyses. To minimize differences in visual stimuli between the baseline and experimental conditions, two identical neutral symbols were presented on the assessment screen, each corresponding to the lightning symbol and monetary information. Since our study specifically focuses on the brain correlates of reward expectation in aggression, we did not establish a corresponding baseline for the decision-making phase. The task’s operational approach was aligned with that of the HGT.

After completing the HGT, participants were fully debriefed and informed that Role B was fictitious and that their actions had not caused harm to any real person.

### 2.3. Moral Disengagement Survey

To test hypotheses 3 and 4, we designed a moral disengagement survey based on prior research [39]. This survey was developed to assess participants’ moral disengagement regarding aggressive decision-making in the HGT. The survey consists of 15 items (e.g., “The electrical shocks used in the experiment are safe, and there is no anticipated significant harm to Role B”), rated on a 5-point Likert scale, where 1 represents “completely inconsistent” and 5 represents “completely consistent.” A higher overall average score indicates a higher level of moral disengagement in the HGT. Participants completed the survey (Chinese version) outside the MRI scanner after finishing the HGT. See the Supplementary Material for complete contents.

### 2.4. fMRI Data Acquisition

Functional images were obtained on a 3T Siemens Tim Trio scanner (Siemens Medical Systems, Erlangen, Germany) with a 12-channel brain array coil. fMRI images were acquired using a T2*-weighted echoplanar BOLD-sensitive sequence with interleaved acquisition (field of view = 220 × 220 mm^2^; acquisition matrix = 96 × 96; voxel size = 3.4 × 3.4 × 4 mm^3^; repetition time (TR) = 2000 ms; echo time (TE) = 30 ms; flip angle = 90°). Each volume comprised 32 axial slices (slice thickness = 3 mm; slice gap = 1 mm), allowing whole brain coverage. For the HGT and symbol assessment task, the average time taken by participants to complete each trial was 17 s and 12 s, respectively (see Figure 1). Therefore, participants spent 1220 s and 360 s completing the HGT and symbol assessment task in the scanner, with a total of 790 BOLD images acquired ((1220 s + 360 s)/TR). To ensure the scanner reached steady-state magnetization, 13 s of gradient and radio frequency pulses preceded each of the two runs in HGT and the symbol assessment task.

Additionally, MPRAGE (magnetization-prepared rapid-acquisition gradient echo) structural images were acquired (TR = 1900 ms, TE = 2.52 ms, inversion time = 900 ms, flip angle = 9°, thickness = 1 mm, number of slices = 176, resolution matrix = 256 × 256 mm^2^, and voxel size = 1 × 1× 1 mm^3^).

All MRI data were collected from the Brain Imaging Center of the Cognitive and Personality Key Laboratory (Ministry of Education), Southwest University, Chongqing, China.

### 2.5. Preprocessing of Imaging Data

Functional images were obtained on a 3T Siemens Tim Trio scanner (Siemens Medical Systems, Erlangen, Germany) with a 12-channel brain array coil.

The imaging data preprocessing was implemented using SPM12 (http://www.fil.ion.ucl.ac.uk/spm/software/spm12/; accessed on 6 December 2019). First, slice order effects were corrected using slice timing correction (acquisition order: ascending; referencing slice: the 31st). Second, head motion correction was implemented to correct head movement artifacts, and the participants who exhibited excessive head motion (3 mm maximum displacement and a 3° rotation throughout scans) were excluded. Third, each participant’s image was spatially normalized to the standard Montreal Neurological Institute (MNI) template with a resampled voxel size of 3 × 3 × 3 mm. Fourth, the data were smoothed with an isotropic 8 mm full width at a half-maximum Gaussian kernel.

### 2.6. Data Analysis

#### 2.6.1. Behavioral Data Analysis

Behavioral data analysis was conducted using SPSS 25 software. A paired-sample *t*-test was employed to examine the differences in participants’ aggressive reward expectation (i.e., subjective satisfaction) and aggressive behavior (i.e., the proportion of choosing electric stimulation) between two monetary conditions in the HGT. This analysis was performed to assess the validity of the HGT’s monetary setting.

#### 2.6.2. fMRI Data Analysis

At the individual level, we constructed three general linear models (GLMs) to analyze the voxel-wise activation during reward expectation phase under the high monetary condition and low monetary condition and the differences in voxel-wise activation between these two conditions in the HGT.

The first GLM was used to examine the voxel-wise activation in reward expectation under the high monetary condition, and in the GLM, we modeled the reward expectation phase and the baseline condition separately. The second GLM was used to examine the voxel-wise activation in reward expectation under the low monetary condition, and in the GLM, we modeled the reward expectation phase and the baseline separately. The third GLM was used to examine the different voxel-wise activation during the reward expectation phase between high and low monetary conditions, and in the GLM, we modeled the reward expectation phase under high and low monetary conditions separately. Six movement parameters were added to the model as nuisance regressors. A canonical hemodynamic response function was convolved at each trial’s onset of the corresponding events. Then, contrast images during the reward expectation phase under the high monetary condition vs. baseline, low monetary condition vs. baseline, and high monetary condition vs. low monetary condition were calculated using GLMs.

At the group level, we conducted a one-sample *t*-test to examine the activation differences during the reward expectation phase among the three conditions: high monetary condition vs. baseline, low monetary condition vs. baseline, and high monetary condition vs. low monetary condition. Multiple comparison correction was performed using the family-wise error (FWE) method (the voxel-level significance threshold was set at *p* < 0.005, uncorrected; the cluster-level significance threshold was set at *p* < 0.05, corrected).

Moreover, we extracted the mean beta value of the significant brain regions during the reward expectation phase (in the contrastive analysis of high vs. low monetary condition) using REX toolbox (http://web.mit.edu/swg/software.htm; accessed on 12 October 2018). Then, Pearson correlation analysis was performed to explore the relationships between these regions and participants’ moral disengagement scores and changes in aggressive behavior (high vs. low monetary condition) in the HGT.

## 3. Results

### 3.1. Behavioral Results

Paired-sample *t*-test analysis revealed that, in the HGT, relative to the low monetary condition, participants exhibited significantly higher satisfaction with the expected reward under the high monetary condition (*M*_high_ = 3.22, *M*_low_ = 1.91, *t* = 7.44, *p* < 0.001). Participants displayed significantly more aggressive behavior (i.e., administering electric stimulation to Role B) under the high monetary condition (*M*_high_ = 0.83, *M*_low_ = 0.39, *t* = 9.1, *p* < 0.001). These findings indicate that the design of the experiment conditions was effective.

### 3.2. fMRI Results

#### 3.2.1. Contrast: Aggressive Reward Expectation Under High Monetary Condition Versus Baseline

**Figure 3:**
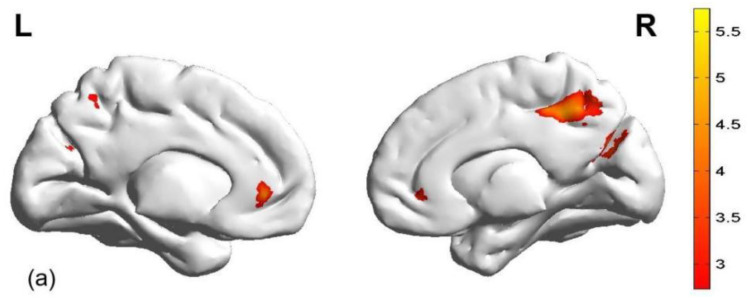

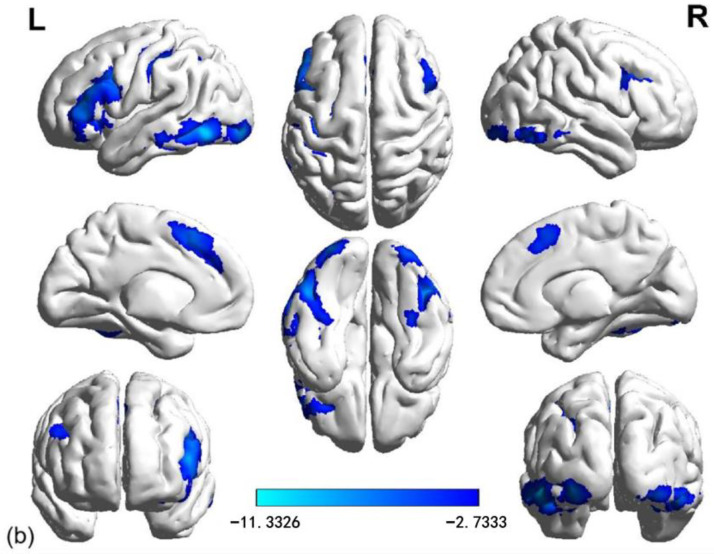
Brain activation during the aggressive reward expectation phase (high monetary condition vs. baseline). (**a**) >baseline; (**b**) <baseline.

Firstly, we explored the brain activation during the reward expectation phase under the high monetary condition relative to the baseline in the HGT. The results showed, relative to the baseline, significant activation in the OFC/ACC, cuneus, and middle cingulate cortex (MCC) during the aggressive reward expectation phase. Conversely, relative to the baseline, activation in the superior frontal gyrus (SFG), inferior frontal gyrus (IFG), postcentral gyrus (PG), IPL, middle frontal gyrus (MFG), inferior temporal gyrus (ITG), sub-gyral, fusiform gyrus, and supplementary motor area (SMA) was significantly decreased during the reward expectation phase (see Table 1 and Figure 3).

#### 3.2.2. Contrast: Aggressive Reward Expectation Under Low Monetary Condition Versus Baseline

**Figure 4:**
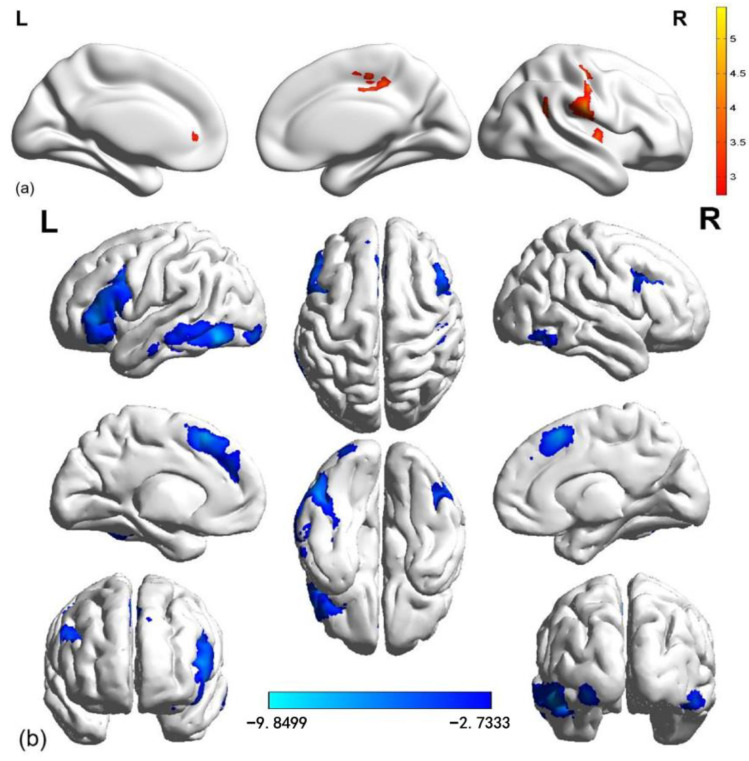
Brain activation during the aggressive reward expectation phase (low monetary condition vs. baseline). (**a**) >baseline; (**b**) <baseline.

Secondly, we examined the brain activation during the reward expectation phase under the low monetary condition, relative to the baseline, in the HGT. The results revealed that, relative to the baseline, the ACC, MCC, right PG, extra-nuclear, and supramarginal gyrus were significantly activated during the reward expectation phase. Conversely, relative to the baseline, activation in the PG, IFG, MFG, ITG, middle temporal gyrus (MTG), and SMA was attenuated during the aggressive reward expectation phase (see Table 2 and Figure 4).

#### 3.2.3. Conjunction Analysis: Overlapping Brain Regions in Aggressive Reward Expectation Under High and Low Monetary Conditions

**Figure 5:**
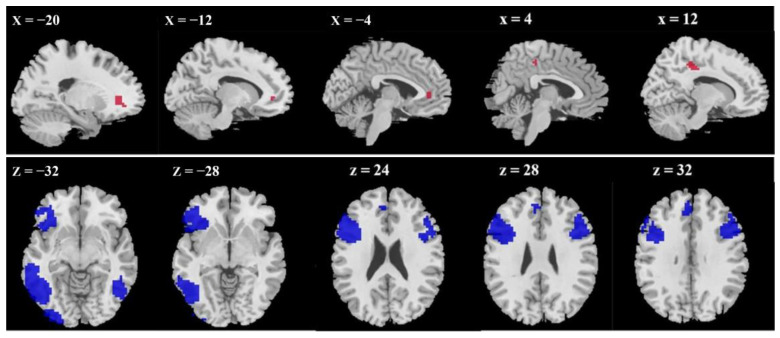
Common areas during the aggressive reward expectation phase under the high and low monetary conditions, relative to the baseline. Notes: Red, >baseline; blue, <baseline.

Furthermore, a conjunction analysis was conducted to investigate the similarity in activation patterns during the reward expectation phase between high and low monetary conditions. The findings showed that, relative to the baseline, common regions that were significantly activated included ACC and MCC. On the other hand, relative to the baseline, common regions where the activation was significantly decreased encompassed the bilateral DLPFC, bilateral lateral temporal cortex (LTC; including ITG and MTG), and SMA (see Figure 5).

#### 3.2.4. Contrast: Brain Activation Differences in Aggressive Reward Expectation Between High and Low Monetary Conditions

**Figure 6:**
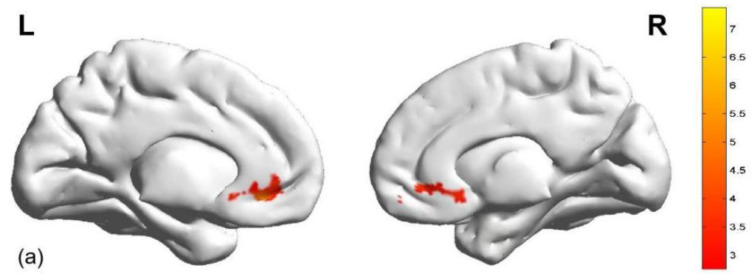

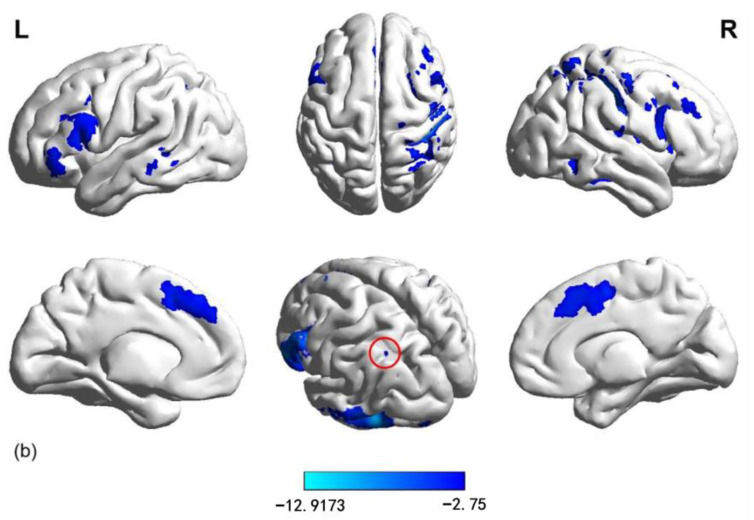
Brain activation during the aggressive reward expectation phase (high vs. low monetary condition). (**a**) >low monetary condition; (**b**) <low monetary condition. Note: The brain region within the red circle is the left IPL.

Thirdly, we examined the differences in activation during the reward expectation phase between the two monetary conditions in the HGT. Relative to the low monetary condition, significant activation in the OFC/ACC was revealed during the reward expectation phase under the high monetary condition. In contrast, activation in the bilateral DLPFC (including the IFG, MFG, and SFG), insula, IPL, bilateral LTC and SMA was decreased during the reward expectation phase under the high monetary condition (see Table 3 and Figure 6).

#### 3.2.5. The Relationships Between Brain Activation in Aggressive Reward Expectation and Aggressive Behavior and Moral Disengagement

**Figure 7:**
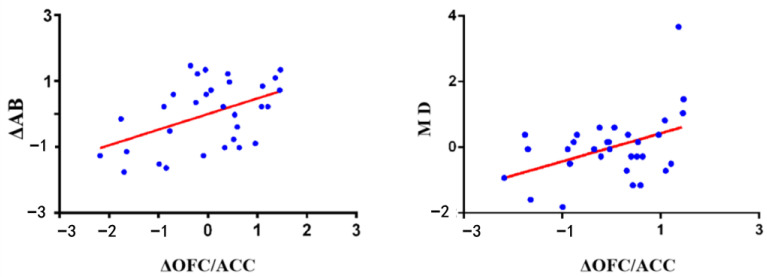
The correlation between changes in OFC/ACC activation during the reward expectation phase (high vs. low monetary condition) and ΔAB and moral disengagement scores in the HGT. Notes: Δ = a mathematical symbol denoting the degree of change; AB = aggressive behavior; MD = moral disengagement; OFC = medial orbitofrontal cortex; ACC = anterior cingulate cortex.

Finally, we extracted the mean beta values of significant brain regions during the reward expectation phase under the high monetary condition, relative to the low monetary condition. Additionally, we computed the change in aggressive behavior (ΔAB; high vs. low monetary condition) in the HGT for each participant. Pearson correlation analysis revealed significant relationships between the mean beta values in the OFC/ACC and participants’ ΔAB and moral disengagement scores (see Figure 7).

Furthermore, the brain areas significantly activated during the aggressive reward expectation phase (high vs. low monetary condition) involved both the OFC and ACC (peak MNI coordinates: −6, 36, −9; see Table 3 and Figure 6a). Therefore, the mean beta values of areas significantly activated in the OFC and ACC were separately extracted, and we examined their correlations with ΔAB and moral disengagement scores. Refer to Table 4 for more detailed information.

Additionally, prior research indicated that moral disengagement mediates the relationship between aggressive motivational factors and aggression [5]. Thus, we examined the mediation effect of moral disengagement between brain activation during the reward expectation phase and ΔAB in the HGT using the PROCESS macro [50]. The findings indicated that the mean beta values in the OFC/ACC during the reward expectation phase (high vs. low monetary condition) significantly predicted participants’ ΔAB (*β* = 0.37, *SE* = 0.18, *p* = 0.046) and moral disengagement scores (*β* = 0.43, *SE* = 0.17, *p* = 0.016). However, the path coefficient between moral disengagement and ΔAB was not significant (*β* = 0.23, *SE* = 0.18, *p* = 0.21).

## 4. Discussion

Our results revealed that brain correlates of aggressive reward expectation involve the OFC, ACC, MCC, DLPFC, IPL, insula, LTC, and SMA. In the HGT, relative to the baseline, OFC, ACC, and MCC were significantly activated during the reward expectation phase. Conversely, activation in the DLPFC, IPL, insula, LTC, and SMA during the reward expectation phase was significantly lower than the baseline. Furthermore, during the reward expectation phase, as the reward increased (high vs. low monetary condition), activation in the OFC and ACC significantly heightened, while activation in the LPFC, IPL, insula, LTC, and SMA decreased. These findings are largely consistent with Hypotheses 1 and 2. Moreover, during the reward expectation phase, as the reward increased (high vs. low monetary condition), heightened activation in the OFC and ACC predicted participants’ ΔAB and was positively correlated with their moral disengagement. Therefore, Hypotheses 3 and 4 are supported.

Consistent with our hypotheses, the OFC and ACC are key brain correlates of aggressive reward expectation. Many studies indicated that the OFC is a critical region for encoding the value of expected reward [24,26,28,29] and is related to the motivational influence of expected reward on behavior [26,35]. In the HGT, relative to the baseline, the OFC was significantly activated during the reward expectation phase only under the high monetary condition. This might be because participants’ satisfaction was lower for expected smaller rewards, which may not induce a significant activation in the OFC. Furthermore, as participants’ satisfaction with the expected rewards increased (high vs. low monetary condition), there was a higher level of activation in the OFC, thereby promoting participants’ aggressive behavior. Overall, the results indicate that the OFC is closely associated with representing the value of expected aggressive reward, suggesting that the OFC may constitute a critical neural basis of aggressive motivation.

The ACC is critical for processing cognitive conflict [44,45,46]. Previous research indicated that when participants face a conflict between aggressive drivers and moral standards, the ACC exhibits significant activation [13,51]. Our results revealed significant activation in the ACC during the aggressive reward expectation phase relative to the baseline, and the level of activation increased with higher expected rewards (high vs. low monetary condition). Expected reward is an important motivational factor in triggering aggressive intention [13,38], which is typically viewed as morally unacceptable [40,41]. In other words, during the reward expectation phase in the HGT, participants were likely to experience a moral conflict. Therefore, significant activation in the ACC during the aggressive reward expectation may reflect a conflict between aggressive inclination and the inhibition of moral system. Furthermore, as anticipated benefits increase, the conflict may intensify. This may explain significant increased activation in the ACC under the high monetary condition in the HGT. Additionally, moral disengagement is closely associated with moral conflict, and is considered a cognitive strategy to cope with moral conflicts [5,13,39]. Thus, in the HGT, participants’ moral engagement can be seen as a criterion variable for moral conflict in aggressive reward expectation. The present study found that, in the HGT, increased ACC activation during the aggressive reward expectation phase (high vs. low monetary condition) was positively correlated with participants’ moral disengagement. In conclusion, our findings imply that the ACC is a critical brain correlate in the process of aggressive reward expectation, and it likely closely relates to the moral conflict involved in this process.

Conjunction analysis revealed that, relative to the baseline, the MCC was significantly activated during the aggressive reward expectation phase in both monetary conditions. Nonetheless, there was no significant difference in MCC activation between the high and low monetary conditions. It is implied that MCC might not be involved in reward processing during the aggressive reward expectation. Previous research indicated that the MCC is linked to self-appraisals or self-identity [52,53] and empathy for pain [54]. Therefore, the MCC may be related to moral inhibition in the aggressive reward expectation. However, unlike other brain regions associated with moral inhibition (e.g., DLPFC), the activation level of MCC was significantly higher during the aggressive expectation phase relative to the baseline. Clearly, the role of MCC in the aggressive reward expectation requires further investigation.

During the aggressive reward expectation phase in the HGT, activation in the bilateral DLPFC, IPL, insula, LTC, and SMA was lower than that in the baseline. Moreover, activation in these brain regions was significantly decreased under the high monetary condition relative to the low monetary conditions. According to Cognitive Neoassociation Theory [36,37], the mental components in the aggression schema are interconnected. Therefore, under the driving influence of expected aggressive reward, brain regions associated with moral inhibition may be recruited. The DLPFC and SMA are closely related to behavioral control, moral judgment, and moral emotions, playing an important role in moral inhibition [13,55,56,57,58]. The IPL is a part of the default network and involves theory of mind, empathy, and moral judgments [18,59,60]. The insula is an essential component of the aversive network and is linked to moral emotions (e.g., guilt) [55,61]. For example, the insula exhibits significant activation when anticipating engagement in unethical behavior or experiencing guilt [13,62]. Thus, it can be inferred that the expected reward for aggression may attenuate moral inhibition, and as the magnitude of the reward increases, the moral inhibition is likely to be further diminished. Additionally, LTC is linked to numerous psychological processes, such as theory of mind [63], semantic memory [64], and moral cognition [19]. Previous research also showed that attenuated response in the LTC is related to impaired inhibitory control [65]. Therefore, in aggressive reward expectation, LTC may be involved in speculating about the victim’s experiences and retrieving moral standards stored in memory. In other words, LTC may be associated with moral inhibition in aggressive reward expectation. To sum up, our results suggest that brain regions involved in moral inhibition are closely linked to aggressive reward expectation.

Although the results largely confirm our hypotheses, some of our hypotheses are not supported. Specifically, we did not find significant involvement of the striatum and regions associated with episodic future thinking in the default network during the aggressive reward expectation phase. This discrepancy may be linked to the task paradigm utilized in the present study. The striatum is associated with learning behaviors, such as forming cognitive connections between actions or stimulus signals and rewards [22,23]. However, in the HGT, participants’ mental processing primarily centers on evaluating the expected reward, rather than predicting potential rewards in response to cues. This could be the main factor contributing to the absence of significant striatum activation in the HGT. Additionally, the HGT does not involve complex cognitive processing. Thus, in the HGT, although aggressive reward expectation involves future-oriented cognition, it does not require detailed or vivid imagination. This indicates that participants may not need to retrieve information from the memory system to generate specific future scenarios or events. Therefore, we did not observe significant activation in brain regions associated with future imagination, such as the hippocampus or posterior cingulate cortex, during aggressive reward expectation.

Although the findings largely align with our hypotheses, it is important to note that the interpretations of these results are correlational inferences. We proposed that aggressive reward expectation involves four primary mental components: episodic future thinking, reward processing, moral inhibition, and moral conflict. However, in the HGT, only reward processing is characterized by an external measurement indicator, namely, participants’ satisfaction with the expected reward. Thus, this study lacks direct evidence that the brain correlates of aggressive reward expectation are associated with episodic future thinking, moral inhibition, and moral conflict. While we use moral disengagement as a criterion variable for moral conflict, which is considered closely related to moral conflict [5,39,48], it does not fully represent the entirety of moral conflict. Therefore, we suggest that future research should develop questionnaires or interview sections specifically designed for the HGT to investigate participants’ moral inhibition, moral conflict, and imagining reward acquisition in aggressive reward expectation. Subsequently, the relationships of these mental components with the brain correlates of aggressive reward expectation can be examined.

Furthermore, this study contributes to the clinical intervention of aggressive behavior. The findings indicate that specific cortical areas, such as the DLPFC and IPL, are associated with reduced aggressive reward expectations. This suggests that applying external interventions, such as transcranial direct current stimulation (tDCS), to these cortical regions may modulate the cognitive processing of anticipated aggressive rewards, thereby reducing aggressive behavior. In conclusion, our research offers theoretical insights and guidance for neurologically based cognitive interventions aimed at mitigating aggressive behavior.

## 5. Limitations and Future Directions

There are a few limitations in this study. Firstly, we exclusively investigated the brain correlates of monetary reward expectation in aggression. However, aggressive behavior may also yield potential social benefits or positive emotional experiences. Thus, future research should explore the brain mechanisms underlying other forms of aggressive reward expectation. Secondly, our study only investigates the brain correlates of reward expectation in proactive aggression. While reward expectation is considered a primary driving factor for proactive aggression [66,67], it may also facilitate reactive aggression [3,68]. Thus, investigating the brain correlates of reward expectation in other types of aggression, such as reactive aggression, is both necessary and valuable. Thirdly, the findings are based on a limited sample of Chinese university students, and its replicability in other populations and cultural contexts needs further examination. Fourthly, we only explored the brain correlates of the aggressive reward expectation within a controlled laboratory condition. Further research is needed to delve into its neural basis at the trait level. Finally, future studies should aim to provide direct evidence for the mental components of aggressive reward expectation, and further examine their associations with brain correlates of aggressive reward expectation.

## 6. Conclusions

In the field of aggression research, while aggressive reward expectation has received continued attention from scholars, our understanding of its neural correlates remains insufficient. This deficiency hinders the exploration of the biological underpinnings of human aggression from a cognitive perspective. A lack of effective and convenient experimental tasks is a key obstacle in investigating the neural basis of aggressive reward expectation.

The present study, utilizing the HGT, preliminarily revealed the brain correlates of aggressive reward expectation and examined their predictive role in aggressive behavior. Our results indicated that the OFC, ACC, MCC, DLPFC, IPL, and LTC are key brain regions involved in aggressive reward expectation. Furthermore, in the HGT, as the reward increased, enhanced responses in the OFC and ACC during the reward expectation phase were significantly correlated with participants’ aggressive behavior and moral disengagement. This suggests that the OFC and ACC may be linked to the motivating effect of reward expectation on aggressive behavior.

To sum up, the present study reveals that brain areas related to reward processing (e.g., OFC), conflict monitoring (e.g., ACC), and inhibitory control (e.g., DLPFC) are neural correlates of aggressive reward expectation, providing new neurobiological markers for this variable. Since reward expectation drives aggression, this work also furthers our understanding of the neural basis of human aggressive motivation. Additionally, the HGT developed in this study serves as an effective tool for further exploration of brain activation features of aggression.

## Figures and Tables

**Table 1:** Brain activation during the aggressive reward expectation phase (high monetary condition vs. baseline).

Regions	Peak MNI Coordinates			Peak t-Value	Cluster Size
	*x*	*y*	*z*		
>baseline					
OFC/ACC	0	42	3	4.747	257
L_Cuneus	−12	−12	24	3.895	132
R_MCC/Precuneus	9	−33	39	5.745	557
<baseline					
L_SFG/SMA	−3	24	57	−6.673	459
L_IFG/IPL/PG	−48	33	15	−8.631	2263
R_MFG	48	33	18	−5.783	308
L_ITG/Fusiform Gyrus	−51	−60	−15	−11.333	1281
R_ITG/Sub-Gyral/ Fusiform Gyrus	48	−57	−15	−7.86	1231

Notes: L = left; R = right; OFC = orbitofrontal cortex; ACC = anterior cingulate cortex; MCC = middle cingulate cortex; IFG = inferior frontal gyrus; MFG = middle frontal gyrus; SFG = superior frontal gyrus; PG = postcentral gyrus; IPL = inferior parietal lobule; ITG = inferior temporal gyrus; SMA = supplementary motor area.

**Table 2:** Brain activation during the aggressive reward expectation phase (low monetary condition vs. baseline).

Regions	Peak MNI Coordinates			Peak t-Value	Cluster Size
	*x*	*y*	*z*		
>baseline					
L_ACC	−15	42	−3	5.032	91
R_MCC	6	−21	45	5.242	147
R_PG	33	−15	60	4.721	399
R_Extra-Nuclear	30	−12	18	5.467	271
R_Supramarginal Gyrus	63	−54	33	4.356	143
<baseline					
L_IFG/Insula	−48	33	12	−7.11	1337
R_PG/IPL	51	−21	48	−5.499	255
L_ITG/MTG	−51	−60	−15	−9.85	1199
R_ITG	51	27	30	−6.391	328
R_MTG	51	−54	−15	−5.483	274
L_SMA	0	21	54	−8.088	491
R_Cerebellum	12	−81	−27	−6.432	176

Notes: L = left; R = right; ACC = anterior cingulate cortex; MCC = middle cingulate cortex; IFG = inferior frontal gyrus; ITG = inferior temporal gyrus; MTG = middle temporal gyrus; PG = postcentral gyrus; IPL = inferior parietal lobule; SMA = supplementary motor area.

**Table 3:** Brain activation during the aggressive reward expectation phase (high vs. low monetary condition).

Regions	Peak MNI Coordinates			Peak t-Value	Cluster Size
	*x*	*y*	*z*		
High > Low					
OFC/ACC	−6	36	−9	7.378	208
High < Low					
L-IFG/MFG/Insula	−51	15	6	−5.998	596
R-IFG	54	9	33	−5.842	265
L-IPL	−30	−66	39	−3.733	118
R-PG/MFG/SFG/IPL/ Insula/SMA	54	−18	48	−12.917	2720
L-MTG	−69	−51	3	−4.17	259
R-ITG	54	−45	−9	−4.453	177

Notes: L = left; R = right; OFC = orbitofrontal cortex; ACC = anterior cingulate cortex; IFG = inferior frontal gyrus; MFG = middle frontal gyrus; SFG = superior frontal gyrus; IPL = inferior parietal lobule; PG = postcentral gyrus; ITG = inferior temporal gyrus; MTG = middle temporal gyrus; SMA = supplementary motor area.

**Table 4:** Relationships between brain activation in aggressive reward expectation (high vs. low monetary condition) and ΔAB and MD scores in the HGT.

Regions	ΔAB		MD	
	*r*	*p*	*r*	*p*
OFC/ACC	0.47 **	0.007	0.43 *	0.016
only_OFC	0.41 *	0.022	0.37 **	0.039
only_ACC	0.47 **	0.008	0.44 *	0.012
L-DLPFC/Insula	−0.24	0.195	−0.18	0.341
R-IFG	−0.22	0.237	0.01	0.951
L-IPL	−0.08	0.687	0.05	0.797
R-PG/DLPFC/IPL/ Insula/SMA	0.06	0.739	0.16	0.393
L-MTG	0.07	0.335	−0.08	0.522
R-ITG	−0.09	0.401	0.13	0.674

Notes: ΔAB = changes in aggressive behavior (high vs. low monetary condition); MD = moral disengagement; L = left; R = right; OFC = orbitofrontal cortex; ACC = anterior cingulate cortex; DLPFC = dorsolateral prefrontal cortex; IFG = inferior frontal gyrus; IPL = inferior parietal lobule; PG = postcentral gyrus; MTG = middle temporal gyrus; ITG = inferior temporal gyrus; SMA = supplementary motor area. * *p* < 0.05, ** *p* < 0.01.

## Data Availability

Upon reasonable request, the research data can be obtained by contacting the corresponding author. Due to the expensive cost of collecting research data and the involvement of participants’ personal information, the data cannot be made publicly available.

## Supplementary Materials

The following supporting information can be downloaded at: https://www.mdpi.com/article/10.3390/brainsci15121326/s1, Supplementary Material: The survey presented some thoughts regarding causing harm to Role B in Harm-Gain Task. Based on your real-time thoughts during the task, please rate how accurately the following descriptions represent your true thoughts. “①” denotes “completely inconsistent” and “⑤” denotes “completely consistent,” with higher numbers indicating a higher level of concordance. If you did not have the following thoughts at all during the experiment, or if your real thoughts were diametrically opposed to the ones described below, please choose “completely inconsistent”.
