# Supplementary material for: Brain Activation Features in Response to the Expectation of Receiving Rewards Through Aggression
Source: Brain Sci. 2025 Dec 12;15(12):1326. doi: 10.3390/brainsci15121326 (PMC12731093; doi:10.3390/brainsci15121326)
Supplement: Supplementary file 1 [file brainsci-15-01326-s001.zip › Supplementary.pdf]

The survey presented some thoughts regarding causing harm to Role B in Harm-Gain Task. Based on your real-time thoughts during the task, please rate how accurately the following descriptions represent your true thoughts. "①" denotes "completely inconsistent" and "⑤" denotes "completely consistent," with higher numbers indicating a higher level of concordance. If you did not have the following thoughts at all during the experiment, or if your real thoughts were diametrically opposed to the ones described below, please choose "completely inconsistent."

**1. Administering shocks to Role B to obtain money was necessary for the authenticity and effectiveness of the experiment.**
completely inconsistent completely consistent<br>1 2 3 4 5
**2. It is inherent in human nature to pursue personal gain, so there is no moral wrongdoing in opting to administer shocks to Role B for money.**
completely inconsistent completely consistent<br>1 2 3 4 5
**3. Administering shocks was simply intended to provide Role B with a novel experience.**
completely inconsistent completely consistent<br>1 2 3 4 5
**4. Compared to those who chose to administer shocks almost exclusively, my frequency of shock administration was relatively low.**
completely inconsistent completely consistent<br>1 2 3 4 5
**5. Compared to acts of violent crime, the choice to administer shocks to Role B in the experiment is negligible.**
completely inconsistent completely consistent<br>1 2 3 4 5
**6. If Role B experiences physical or psychological discomfort due to the shocks, the primary responsibility lies with the experimenter and Role B. This is because the task was designed by the experimenter, and Role B also accepted this arrangement.**
completely inconsistent completely consistent

1 2 3 4 5

7. I administered shocks to Role B of my own volition, following the instructions provided by the experimenter.

completely inconsistent completely consistent

1 2 3 4 5

8. In this experiment, other participants also administer shocks to role B, so my choice to administer shocks to role B is justified.

completely inconsistent completely consistent

1 2 3 4 5

9. The electrical shocks used in the experiment are safe, and there is no anticipated significant harm to Role B.

completely inconsistent completely consistent

1 2 3 4 5

10. Due to the low intensity of the electrical shock, Role B is not expected to experience significant discomfort in their left wrist after the shock.

completely inconsistent completely consistent

1 2 3 4 5

11. The strength of the electrical shocks used in the experiment is unlikely to cause discomfort to Role B.

completely inconsistent completely consistent

1 2 3 4 5

12. The remuneration provided to Role B for their participation in the experiment effectively compensates for the adverse experiences of receiving electric shocks.

completely inconsistent completely consistent

1 2 3 4 5

13. As Role B chose to participated in the experiment for remuneration, he/she has a duty to acknowledge and assume the risk associated with receiving electrical shocks.

completely inconsistent completely consistent

	1	2	3	4	5	
**14. I didn’t pick electric shocks for every round, so if role B drew the outcome of being shocked, it was purely due to his/her bad luck.**						
completely inconsistent	1	2	3	4	5	completely consistent
**15. Even though I made some decisions to choose electric shocks during the experiment, Role B still might not draw the option of being shocked.**						
completely inconsistent	1	2	3	4	5	completely consistent
